# Effects of nurse-led hierarchical management care on acute stroke patients: A pilot study to promote stroke-associated pneumonia management

**DOI:** 10.3389/fneur.2023.1121836

**Published:** 2023-04-12

**Authors:** Dongxiang Zheng, Shengjuan Li, Yan Ding, Huaihua Chen, Dong Wang, Huan Wang, Yuyao Xie, Chen Li, Jinglan Luo

**Affiliations:** ^1^Department of Neurology and Stroke Center, First Affiliated Hospital of Jinan University, Guangzhou, Guangdong, China; ^2^School of Nursing, Jinan University, Guangzhou, Guangdong, China; ^3^Department of Neurology, The First Affiliated Hospital, Jinan University, Guangzhou, China; ^4^Dapeng New District Nan'ao People's Hospital, Shenzhen, China; ^5^Department of Internal Medicine, First Affiliated Hospital of Jinan University, Guangzhou, Guangdong, China

**Keywords:** ischemic stroke, hierarchical management care, stroke-associated pneumonia, nursing care, pilot study

## Abstract

**Background:**

Stroke-related pneumonia (SAP) is a common complication in acute ischemic stroke (AIS) patients, and it has adverse effects on the clinical outcomes and increases the burden on patients' families and society. Early identification and individualized care are necessary to reduce the incidence of SAP.

**Objective:**

The present study aimed to explore the effect of nurse-led hierarchical management care based on the acute ischemic stroke-associated pneumonia score (AIS-APS) scale in AIS patients.

**Methods:**

A quasi-intervention pilot study design was adopted for the present study. A total of 120 AIS patients were enrolled and assigned to the intervention group and the control group, with 60 subjects in each group in a tertiary hospital in Guangzhou, China. The control group received routine care, whereas the intervention group was given nurse-led hierarchical management care based on the AIS-APS scale. The intervention duration was more than 7 days, and the incidence of SAP, neurological function, swallowing function, and activities of daily living (ADLs) at discharge were observed. The outcomes were assessed at baseline and at outpatient time.

**Results:**

A total of 120 participants were enrolled in our study. A significant decrease was found in the incidence of SAP in the intervention group (18.3%) compared with that in the control group (41.7%). Positive outcomes were shown in neurology function, swallowing function, and ADL in the intervention group.

**Conclusion:**

Nurse-led hierarchical management care based on AIS-APS can reduce the incidence of SAP, promote AIS patients' neurological function, and maintain patients' ADL. The results of our study indicated that nurse-led hierarchical management care is feasible for AIS patients and provides individualized interventions for patients with different levels of SAP risk. Nurse-led hierarchical management care could be incorporated into routine nursing practice. Further study is needed and expected to solve more clinical problems.

## Introduction

Stroke is an acute cerebrovascular disease that is caused by an ischemic infarction or an intracranial hemorrhage (1). Acute ischemic stroke (AIS) is the leading common type of stroke and accounts for 69.6–70.8% of all strokes (2). AIS with the characteristics of high morbidity, disability, mortality, and recurrence, is one of the main diseases that threatens human life and health (1, 3). AIS is accompanied by impaired consciousness, dysphagia, hemiparesis, and stroke-associated pneumonia (SAP).

The concept of SAP was introduced by Hilker et al. (4). The 2010 Chinese Expert Consensus on the Management of SAP defines it as an inflammation of the lung parenchyma (including the alveolar wall or the interstitial lung in the broader sense) in stroke patients who have not previously had pneumonia. When SAP occurs within 72 h of admission, it is described as an early stage of SAP. Another classification that is being used classifies SAPs into acute (when pneumonia occurs within 1 month after stroke) phase and chronic phase (when pneumonia occurs 1 month later) (5). Other SAPs include pendant pneumonia caused by poor blood circulation, long-term bedrest, and ventilator-associated pneumonia (VAP) induced by ventilator-assisted respiration (6).

Stroke-related pneumonia is a common poststroke complication, with a prevalence of ~7–38%, and is the leading cause of death in patients in the acute phase, with a 30-day mortality rate of up to 30% (4, 7, 8). SAP is associated with prolonged hospitalization, delayed recovery, difficulty in performing rehabilitation procedures, poorer functional outcomes, higher mortality, and increased financial and nursing burden on patient families (9, 10). Previous studies have shown that SAP can be prevented (11, 12). Therefore, for AIS patients, it is of important clinical significance to accurately predict the risk of SAP and implement effective prevention and control measures in a timely manner.

Currently, some studies have focused on pneumonia using pharmaceutical interventions (13, 14) or invasive treatments, such as a portable fibrobronchoscopic treatment and tube feeding methods (15, 16), respiratory muscle training (17), or standard care bundle intervention in preventing the occurrence of SAP (18). A common limitation of these studies is that they provide a uniform intervention without assessing patients' SAP risk. Comprehensive measures and individualized interventions are needed to reduce the incidence of SAP.

Hierarchical management care involves targeted nursing interventions according to the severity of the patient's condition to ensure that each nursing measure is more suitable for the patient's condition and improves the quality of nursing (19, 20). Recent researchers have shown that hierarchical management methods have promising results in acute cerebral infarction, acute myocardial infarction, and bronchial asthma (21–23), which have attracted researchers' interest in exploring more potential effects.

The acute ischemic stroke-associated pneumonia score (AIS-APS) (8) was developed on the basis of data from the National Stroke Registry of China. One study (24) showed that the AIS-APS scale has good predictive discrimination and accuracy and is an operational tool that can stratify the SAP risk to quickly screen high-risk patients.

In this study, we aimed to explore the effect of nurse-led hierarchical management care based on the AIS-APS scale on patients with AIS. By refining the level of nursing required for different degrees of AIS, a nursing-level classification scheme was implemented to ensure that each nursing intervention is more suitable for the patient's condition and thus provides a reference for clinical nursing.

The primary hypothesis is stated as follows. After the intervention, a lower incidence of SAP in AIS patients was observed in the intervention group.

The secondary hypothesis is stated as follows. After the intervention, the intervention group would show significant improvements in neurological function, better recovery of the swallowing function, and a prognostic quality of life.

By refining the nursing levels of high-risk patients, the nurse-led hierarchical management care based on the AIS-APS scale was implemented to ensure that each nursing measure is more suitable for the patient's condition and thus provides a reference for clinical nursing specialists to prevent SAP.

## Materials and methods

### Study design and population

We conducted a single-blind non-randomized controlled trial to test our hypothesis. The study was conducted at the First Affiliated Hospital of Jinan University, China, from January 2021 to December 2021. The participants were divided into the intervention group and the control group according to the admission time. The researchers in charge of the outcome evaluation were blinded to the allocation of participants.

The preexperimental results of this study showed that the incidence of SAP in the control group was ~50% and the incidence of SAP in the intervention group was ~20%. According to the sample size calculation formula of the two groups of an equally parallel 1:1 design, by taking α = 0.05 and β = 0.1, the sample size needed for this study was estimated with the following formula (25):


$$\begin{array}{l} n\;=\;\frac{{\text{p}}_{1}\;\times \;\left(1\;-\;{\text{p}}_{1}\right)\;+\;{\text{p}}_{2}\;\times \;\left(1\;-\;{\text{p}}_{2}\right)}{{\left(\text{p}1-\text{p}2\right)}^{2}}\times {\left(\mu_{\alpha /2}+\mu_{\beta }\right)}^{2} \end{array}$$

p1 = 50%, P2 = 20%, α = 0.05, β = 0.1, and substitution into Eq.


$$\begin{array}{l} \frac{0.5\;\times \;\left(1\;-\;0.5\right)\;+\;0.2\;\times \;\left(1\;-\;0.2\right)}{{\left(0.5\;-\;0.2\right)}^{2}}\;\times \;{\left(1.96\;+\;1.28\right)}^{2}\;\approx \;48 \end{array}$$

According to the aforementioned calculation, each group needs at least 48 patients, considering the dropout rate of 10%, and each group should have collected at least 53 AIS patients to ensure that the study can be carried out.

The participants were recruited by word of mouth and posters. The inclusion criteria for the present study were as follows: (1) should have met the diagnostic criteria in the Guidelines for Acute Ischemic Stroke Treatment (26); (2) should have been admitted within 72 h of stroke; (3) should have been admitted without the diagnoses of pulmonary infection; (4) should have an age ranging ≥18 years; and (5) should have the length of the intervention of no < 7 days. The participants were excluded from the present study, if they had (1) been discharged or died within 24 h; (2) had complicated infectious diseases and lung tumors before admission or other respiratory diseases; (3) had incomplete clinical data; or (4) had severe heart, lung, liver, kidney disease, or malignant tumors.

### Ethical considerations

All the participants provided written informed consent prior to the study.

### Intervention methods

#### Intervention group

The team was a multidisciplinary medical, nursing, and technical cooperation team that was composed of an expert group and a research group. The expert group included two neurology clinicians, eight clinical nurses, and one rehabilitation therapist from the neurology intensive care unit, and the research group included one doctor and four nursing master candidate students. The expert group was mainly responsible for the implementation of the interventions and the guidance and supervision of the nursing program, whereas the research group was responsible for the intervention preparation, data collection, and analysis of the findings.

#### Determination of the hierarchical management care

The included participants were classified into Groups I–III according to the AIS-APS scale. The AIS-APS scale includes 11 indicators in 7 domains: (1) age: 0 for ≤ 59 years, 2 for 60–69 years, 5 for 70–79 years, and 7 for ≥80 years; (2) medical history/comorbidity (including five indicators): atrial fibrillation, congestive heart failure, chronic obstructive pulmonary disease, and smoking; (3) prestroke dependence; (4) the National Institutes of Health Stroke Scale (NIHSS) score range at admission was assigned as follows: 0–4 for 0, 5–9 for 2, 10–14 for 5, and ≥15 for 8; (5) the Glasgow Coma Scale (GCS) at admission was assigned as follows: 15–13 for 0, 9–12 for 0, and 3–8 for 3; (6) dysphagia for 3; (7) stroke staging: 0 for lacunar infarction, 0 for partial anterior circulation infarction, 2 for complete anterior circulation, and 2 for posterior circulation infarction; and (7) admission blood glucose level. The total scale score of AIS– APS was 35, and according to the results of the AIS-APS scale, the patients were assigned to Group I: low-risk group (0–13 points), II: medium-risk group (14–20 points), or III: high-risk group (21–35 points).

#### Intervention measures

The intervention lasted from the day of recruitment until day 7 of hospitalization or the day of discharge (in case the patient was hospitalized for more than 7 days). Based on the results of the AIS-APS scale at admission, the AIS-APS hierarchical care was provided by our expert group accordingly, while nurses in charge and supervising physicians dynamically adjusted the nursing care level according to patients' condition during hospitalization. AIS-APS hierarchical care details are shown in Table 1.

**Table 1:** Nurse-led hierarchical management care based on APS-AIS scale.

**Evaluation at admission**	Extremely low-risk stratum □ Smooth stratum□ Intermediate risk stratum□ High risk stratum□ Very high-risk stratum□	
**Care measures**		
**Environment management**	1. Open windows for ventilation twice a day, for 30 min each time	
	2. Room temperature 20°C−22°C, humidity 50–60%	
	3. Limit the number of visitors and the visit duration	
	4. Use chlorine-based disinfectant to clean equipment, bed units, and ward floors	
	5. Patients diagnosed with multidrug-resistant bacteria are provided bedside isolation or single-room isolation	
**Posture management**	1. Assist bedridden patients in attaining proper posture and regularly turn them over and pat their backs	
	2. Encourage patients to use healthy limbs to assist passive movement of affected limbs, such as fork-grip lift training and lower limb bridge exercises	
	3. When the patient is sitting, put both upper limbs on the platform or bedside mobile table, sit firmly and push the patient back and forth alternately; do not make the patient fall down	
	4. Those who have difficulty standing need to do stand-up training first, and start walking training when the patient is able to stand without fatigue.	
	**Dietary management**	**Airway management**
**Extremely low-risk stratum** **Smooth stratum 0–13**	**Patients with water swallow test Level I , Patients with consciousness** 1. Instruct patients to eat through the mouth and correct irrational eating behaviors 2. Create a relatively quiet eating environment	1. Patients should rinse their mouths carefully and brush their teeth well after eating 2. Select a mouthwash according to the patient's oral condition
**Intermediate risk stratum 14–20**	**With the abovementioned nursing measures, add: (Patients with water swallow test Level II , Patients with consciousness)** 1. According to the V-VST screening results and the condition, patients were given a feeding plan with different food consistency (low, medium, and high consistency) and bite size of 5**–**20 mL 2. For patients who choke on water alone, use rennet to thicken liquids (juice, milk, tea, soup, etc.) to reduce the chance of accidental aspiration and choking. 2. Patients were moved into a semirecumbent or sitting position while eating and, after the meal, patients maintained the eating position for 20**–**30 min before returning to the supine position. 3. Hanging “Prevent Aspiration” warning sign on the bedside	**With the above mentioned nursing measures, add:** **Patients who eat** ***via*** **mouth:** 1. Remove oral secretions and food residues before eating 2. Instruct patients to properly cough and excrete sputum before eating, observe whether patients choke and cough up sputum during and after eating, and whether the sputum contains food particles. 3. Cooperate with rehabilitation practitioners to guide patients to perform swallowing function training, such as mouth opening exercise, empty swallowing, and masticatory muscle exercise **Patients receiving nasal feeding:** 1. Oral care 2 times/day 2. During the nasogastric feeding process, observe whether there is choking, dyspnea, nausea and vomiting, etc. If any of the above occurs, stop the nasogastric feeding immediately. 3. Replace nasogastric fluid containers and medication utensils with each meal 4. Wash the skin around the intubation and the skin where the tape is fixed daily, and keep it clean and dry
**High-risk stratum Very high-risk stratum 21–35**	**Patients with water swallow test Level III or V, Patients with impaired consciousness:** 1. Choose the right-sized nasogastric tube 2. Continuous head elevation >30° and verification of tube position before and after feeding, assessment of gastric residual volume 3. The temperature is 38°-40° for nasal fluids not exceeding 200 mL at a time or by enteral nutrition pump infusion. 4. Flush the nasal cannula with 30–50 mL warm water before and after nasal feeding. 5. Maintain semi-recumbent position for 60 min after nasal feeding to avoid food-aspiration-related surgeries 6. Check daily for proper fixation of the nasal feeding tube and any changes in the length of placement	**With the above mentioned nursing measures, add:** **Nasal feeding patients:** 1. Oral care 3 times/day 2. Keep the respiratory tract unobstructed, suction sputum according to the needs of the patient, and perform strict aseptic operation (if necessary, increase the oxygen flow before and after suction) 3. Perform airway humidification, and observe the patient's ventilation and changes in breath sounds during the process 4. Monitor patients for signs and symptoms of infection, including unexplained fever, changes in sputum color and properties, and decreased oxygen saturation 5. Observe the patient's consciousness, heart rate, respiration, blood pressure, blood glucose, and water electrolytes
**Health education**	1. Instruct the patient's family to make good food choices: Light, easily digestible food, which is moderately viscous and does not easily remain on the mucous membranes such as egg custard, tofu, etc.	
	2. Encourage the patient to use the healthy hand or the affected hand to wash the face, brush the teeth, eat, change clothes, etc., and insist on practicing button fastening and using the toilet.	
	3. Families are instructed on anti-sucking techniques, recognition of aspiration/choking, proper oral hygiene care methods, simple rehabilitation training methods, and prevention of pulmonary infections.	
	4. Patients are followed up regularly to understand the problems that they encounter and to provide timely guidance and assistance.	

### Control group

Routine nursing interventions were administered upon admission. Participants in the control group received the usual care.

### Outcome evaluations

The measurement tools included general information questionnaires, the National Institute of Health Stroke Scale (NIHSS), the modified Rankin Scale (mRS), the water swallow test (WST), and the ability to perform the Activities of Daily Living (ADL) Scale, which were used for screening patients and assessing the effectiveness of the intervention.

The outcomes were SAP rate, neurological function, swallowing function, and daily life function at discharge time *via* face-to-face interviews by trained research group members.

Two technicians were responsible for the evaluation of the homogeneous comprehensive training received. The research group was blinded to which group the participants belonged to and completed all the measurements.

### Primary outcome measures

#### Stroke-related pneumonia

Chest x-ray examination showed a new or progressive pulmonary infiltration after stroke, combined with two or more of the following clinical symptoms of infection: (1) a temperature ≥38°C; (2) a new cough, sputum, or aggravation of the existing respiratory symptoms, with or without chest pain; (3) solid signs of disease, or pulmonary wet fissure, with leukocytes ≥10 × 10^9^/L or ≤ 4 × 10^9^/L, with or without a nuclear shift to the left. Routine blood and chest DR examinations were performed at admission and at discharge (or at the end of tube feeding). If the patient is hospitalized with a temperature ≥38°C, or if he or she has a new cough, sputum, or wet cracks in the lung, he or she should receive routine blood and chest x-ray immediately.

### Secondary outcome measures

#### Neurological function

Neurological function was assessed using the NIHSS (27) and the mRS (28). The degree of neurological deficits after admission was evaluated using the NIHSS (scale range: 0–42). The total scale scores of < 4, 4–15, and >15 were graded as mild, moderate, and severe, respectively. The mRS was used to assess the degree of dependence on the patient's ability to perform life activities and to determine the degree of disability after a stroke. An mRs scale of 2 is the criterion and cutoff value for classifying whether a stroke patient is disabled after stroke.

#### Swallowing function

The swallowing function was graded according to the WST (29). The purpose of this experiment was to detect aspiration with high precision. In Japan, two methods of 3 ml or 30 ml of water are usually used. We used the 30-ml method to detect aspiration in otherwise normal AIS patients. The sensitivity and specificity of the test were 70 and 71%, respectively. The patients were asked to drink water from a 30-ml glass in their usual manner. Their drinking patterns and their voice changes after drinking were recorded (wet voice). Drinking patterns were defined as follows: Level 1: drink 30 ml of water without choking; Level 2: swallow 30 ml of water multiple times without asphyxia; Level 3: drink 30 ml of water at a time but with a choking sound; Level 4: repeated ingestion of 30 ml of water with asphyxia; and Level 5: choking sound and difficulty in drinking 30 ml of water.

#### Activities of daily living

A comprehensive assessment of 10 items, such as diet, grooming, self-control, and bed chair transfer, was performed using the ADL (30), each rated at 10 out of 100. The higher the scale, the less help the patient needs and the lower the dependency.

### Statistical methods

SPSS 22.0 software was used for conducting statistical analysis. The distribution of data was tested using the Shapiro–Wilk test. Continuous variables that conformed to the normal distribution were described by means and standard deviations (SD), and those that did not conform to the normal distribution were described by medians and quartiles. Categorical data were described in terms of frequency and percentage. Baseline characteristics of the intervention and control groups were compared by independent *t-*tests, nonparametric tests, or chi-squared (x^2^) tests. Data from the control and intervention groups were compared using independent and paired *t*-tests (the differences were normally distributed)/nonparametric tests, respectively. A p-value of two-sided tests of < 0.05 was considered to be statistically significant.

## Results

### Baseline characteristics of participants

**Figure 1:**
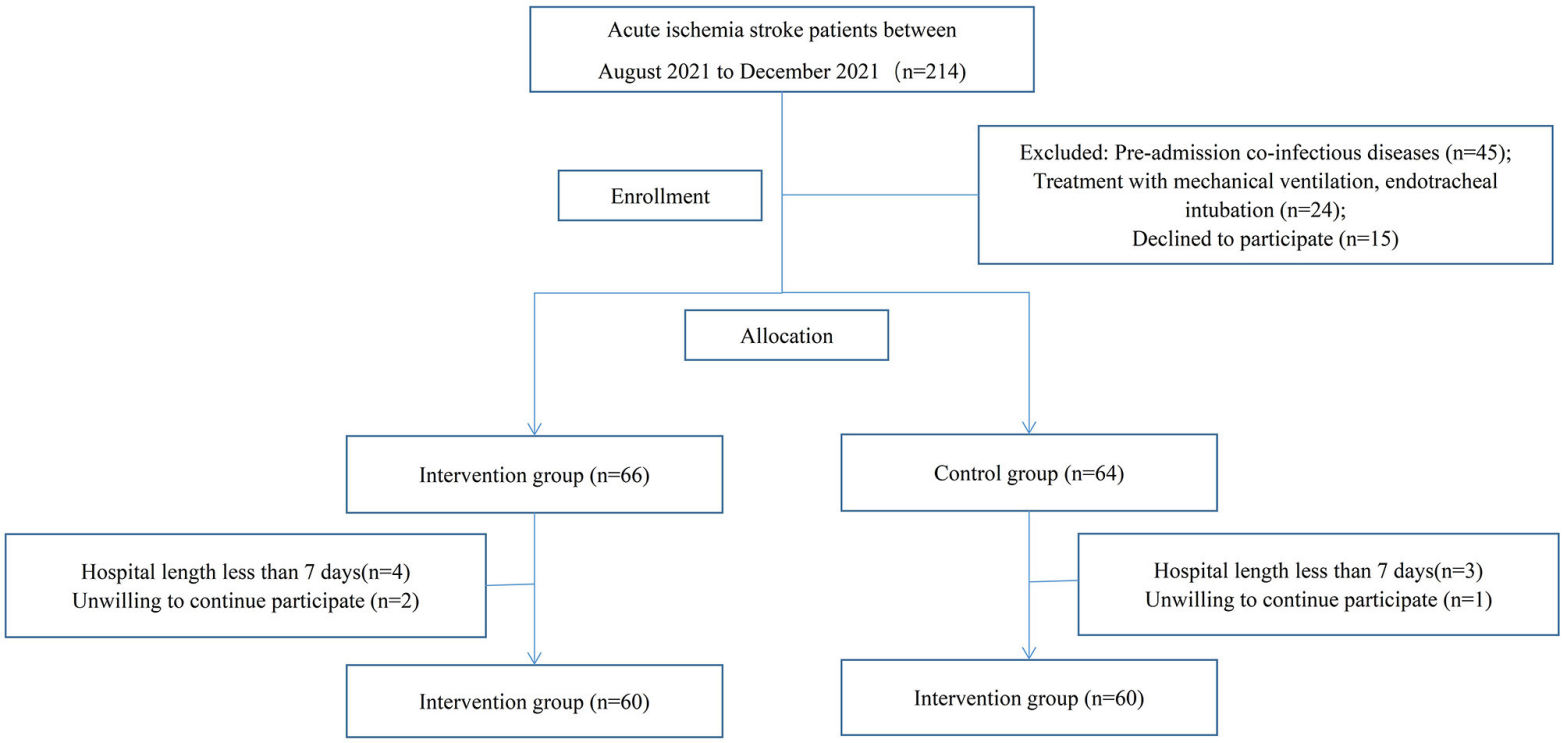
Flowchart showing recruitment and dropout of participants.

The recruitment and dropout details of the participants are shown in Figure 1. As a result, 120 eligible participants were recruited in the study (60 in the nurse-led hierarchical management care group and 60 in the control group).

The age of the participants in the intervention group was 62.70 ± 12.52 years, and 41 patients (68%) were men. The age of the participants in the control group was 66.28 ± 10.02 years, and 43 patients (72%) were men. There were no significant differences in demographic features, namely, gender, age, hypertension, diabetes mellitus, dyslipidemia, atrial fibrillation, current smoking, and excess alcohol consumption (*p*>0.05). However, in the control group, there were more patients with a partial anterior circulation infarct in the OCSP subtype (p>0.05), which means that the patient's condition was more severe (Table 2).

**Table 2:** Baseline characteristics of study participants.

**Characteristics**	**Intervention group (*n =* 60)**	**Control group** **(*n =* 60)**	**t or χ^2^ value**	***P*- value**
Sex			0.444	0.659
Male	41	43		
Female	19	17		
Mean age (years)	62.70 ± 12.52	66.28 ± 10.02	0.898	0.492
Hypertension (*n* %)			1.150	0.255
Yes	33 (55.0 )	38 (63.3 )		
No	27 (45.0 )	22 (36.7 )		
Diabetes mellitus (n, %)			0.753	0.454
Yes	15 (25.0)	19 (31.7 )		
No	45 (75.0)	41 (68.3 )		
Dyslipidemia (n, %)			1.843	0.070
Yes	11 (18.3)	4 (6.7 )		
No	49 (81.7)	56 (93.3 )		
Atrial fibrillation (n, %)			−1.000	0.321
Yes	6 (10.0 )	3 (5.0 )		
No	54 (90.0 )	57 (95.0 )		
History of stroke/TIA (n, %)			−2.256	0.028
Yes	14 (23.3)	10 (16.7)		
No	46 (76.7)	50 (83.3)		
Current smoking (n, %)			1.230	0.224
Yes	20 (33.3)	26 (43.3)		
No	40 (66.7)	34 (56.7)		
Excess alcohol consumption (n, %)			0.574	0.568
Yes	7 (11.7)	5 (8.3)		
No	53 (88.3)	55 (91.7 )		
OCSP subtype, n, %			2.930	0.005
Lacunar infarction	25 (41.7 )	11 (18.3 )		
Partial anterior circulation infarct	13 (21.7 )	16 (26.7 )		
Total anterior circulation infarct	10(16.7)	11 (18.3 )		
Posterior circulation infarct	12(20.0)	22 (36.7 )		
AIS–APS score			−3.984	0.000
0–6	13	20		
7–13	21	17		
14–20	17	15		
21–27	5	5		
28–35	4	3		
mRS scale			2.390	0.020
0	6	5		
1	31	23		
2	22	30		
3	0	0		
4	1	0		
5	0	1		
NIHSS scale	12.97 ± 7.62	9.83 ± 7.53	1.76	0.083

TIA, transient ischemia attack; OCSP, Oxfordshire community stroke project; AIS–APS, acute ischemic stroke-associated pneumonia score.

### Feasibility

The study enrolled participants who were engaged in the intervention during hospitalization. During the intervention period, 10 participants were excluded for intervention duration and personal willingness. All 120 participants completed the intervention program in accordance with a detailed program protocol. In the meantime, expert groups can readily assess the patients' conditions and adjust the nurse-led hierarchical management care level accordingly. Nurses in charge can effectively implement nursing interventions.

### SAP

Stroke-related pneumonia occurred in 11 (18.33%) out of 60 patients in the intervention group and 25 (41.67%) out of 60 patients in the control group. The incidence of SAP in the intervention group was lower than that in the control group, and the differences were statistically significant (p < 0.05) (Table 3).

**Table 3:** The comparisons in outcomes between two groups after intervention.

**Variables**	**Category**	**Intervention group**	**Control group**	**Statistic**	***p*-value**
SAP, *n* (%)	Yes	11 (18.33)	25 (41.67)	7.778	0.005^*^
	No	49 (81.66)	35 (58.33)		
Neurology function	NIHSS score (d, x ± s)	3.10 ± 4.13	4.64 ± 4.93	2.212	0.031^*^
	mRS score			6.001	0.000^*^
	0	34	28		
	1	25	26		
	2	1	5		
	3	0	1		
	4	0	0		
	5	0	0		
Swallowing function (d, x ± s)	/	1.93 ±1.08	1.17 ±0.42	5.520	0.003^*^
ADL (d, x ± s)	/	76.83 ± 20.65	57.13 ± 29.08	4.950	0.000^*^

SAP, stroke-related pneumonia; ADL, activities of daily living; NIHSS, National Institute of Health stroke scale; mRS, modified Rankin Scale. ^*^p < 0.05.

### Neurological function

The results showed that the NIHSS score was 3.10 ± 4.13 in the intervention group and 4.64 ± 4.93 in the control group (p < 0.05), and the mRS score was 1.57 ± 1.21 in the intervention group and 2.84 ± 1.51 in the control group (p < 0.05) (Table 3).

### Swallowing function

After the intervention, the swallowing function in the intervention group was 1.93 ± 1.08, which was a significant improvement compared to the control group of 1.17 ± 0.42 (Table 3).

### ADL

The ADL was higher in the intervention group (76.83 ± 20.65) than in the control group (57.13 ± 29.08), with statistically significant differences (p < 0.05) (Table 3).

### Adverse events

No direct adverse events were observed in the intervention group.

## Discussion

Based on our results, it can be concluded that nurse-led hierarchical management care based on AIS-APS exerts a positive effect on the SAP rate and improves stroke prognosis among AIS patients. Furthermore, the results of the present study suggest that hierarchical management care is a suitable intervention that can be led by clinical nurses because it is feasible and efficient.

Neuroinflammation and stroke-induced immunosuppression contribute to stroke-associated infections, such as SAP (31–33). Various risk factors for SAP have been identified, such as male gender, older age, dysphagia, severe stroke, and disturbance of consciousness (34). According to the APS-AIS, we identified patients with low-risk, medium-risk, and high-risk SAP. AIS-APS hierarchical management care was applied to assess acute ischemic stroke patients admitted within 24 h of stroke, and targeted preventive measures and hierarchical management care were given based on the screening results.

Stroke-related pneumonia is associated with increased long-term mortality and poor functional outcome on discharge (10). With the lower incidence of SAP, lower NIHSS and higher mRS scores were observed. Targeted intervention strategies are required to improve the outcomes of SAP patients who survive to hospital discharge.

Dysphagia puts stroke patients at a higher risk of pneumonia, disability, and death, and early dysphagia screening appears to be associated with a reduced risk of stroke-related pneumonia and disability (33, 35). Compared to the study of Liu, in addition to the measures of position management, feeding management, position, airway, and oral hygiene (18), we also included taking environment management, swallowing function rehabilitation, and diet management in our intervention measures. With dysphagia management and rehabilitation intervention, improvement was shown in the swallowing function. In the meantime, target intervention strategies are beneficial in SAP management.

It is feasible for clinical nurses to perform hierarchical management of care because AIS-APS score was obtained on an operational scale. Before implementation, a clinical care team needs to be established, with members responsible for monitoring and implementing the hierarchical management of care. Training is also needed to teach clinical nurses how to assess the SAP risk using the AIS-APS scale. Hierarchical management care measures require changes according to the evidence-based summary of the reasons for SAP.

### Implications for further study

Nurses play an important role in hierarchical management care, but it is necessary to evaluate the process by which the nurses implement these measures. In the meantime, the implementation process should be supervised for maximum effect.

Acute ischemic stroke caused by a large vessel occlusion is often accompanied by greater stroke severity and a much higher prevalence of SAP (36). For further study, we will narrow down our target population and focus on the AIS patients after vascular revascularization therapy.

The role of clinical risk scores in predicting SAP in clinical care or research may lead to bias (37). A further study should focus on developing a risk prediction model based on subjective indicators, such as blood markers.

### Limitations

The present study had the following limitations. First, the duration of intervention varies depending on the hospitalization time. Whether a longer intervention time can achieve better outcomes remains unknown. The follow-up time was insufficient to determine long-term patient prognosis. Therefore, further study should extend and standardize the intervention duration. Second, the small convenience sample, while appropriate for a feasibility study, did not provide adequate statistical power and limits generalizability to other populations. Further studies should be well designed, and the sample size should be larger to obtain more positive results.

## Conclusion

This pilot study demonstrates that hierarchical management care based on the AIS-APS scale is feasible to be implemented in AIS patients and that it has the potential to decrease the incidence of stroke-related pneumonia and improve patients' prognosis. The present pilot study paves the way for developing hierarchical management nursing programs with generalization and sustainability.

## Data availability statement

The raw data supporting the conclusions of this article will be made available by the authors, without undue reservation.

## Ethics statement

The studies involving human participants were reviewed and approved by First Affiliated Hospital of Jinan University (KY-2021-108). The patients/participants provided their written informed consent to participate in this study. Written informed consent was obtained from the individual(s) for the publication of any potentially identifiable images or data included in this article.

## Author contributions

DZ, CL, SL, and JL made substantial contributions to conception and design. JL, YD, and HC in charge of the intervention implementation. DW, HW, and YX in charge of the intervention preparation and data collection. DZ and SL analyzed the data and prepared figures and/or tables. SL and CL wrote the article. All authors contributed to manuscript revision and approved the submitted version.
